# Automatic Measurement of External Thread at the End of Sucker Rod Based on Machine Vision

**DOI:** 10.3390/s22218276

**Published:** 2022-10-28

**Authors:** Xianyou Li, Shun Wang, Ke Xu

**Affiliations:** 1 Institute of Engineering Technology, University of Science and Technology Beijing, Beijing 100083, China; 2 Department of Mechanical Engineering, Baotou Vocational and Technical College, Baotou 014030, China; 3 Collaborative Innovation Center of Steel Technology, University of Science and Technology Beijing, Beijing 100083, China

**Keywords:** precise measurement, screw thread, sucker rod, machine vision

## Abstract

Aiming at the low efficiency of manual measurement of threads and the lack of practicability in machine vision measurement before, online size measurement of threads at the end of sucker rods based on machine vision was studied. A robotic arm is used to carry an optical device to achieve high-quality image acquisition of threads. Based on the prior knowledge of the thread profile angle, the directional edge detection operator is customized to achieve the accurate detection of the left and right edges of the thread. Noise filtering, sorting, and left and right edge-matching algorithms based on connected domains are developed to eliminate the interference effects of electrostatic dust and oil pollution in online measurement, and the dimension of thread profile angles, pitches, major diameters, and minor diameters can be precisely calculated. The experimental results show that the screw thread parameter measurement time is about 0.13 s; the maximum and minimum average errors of the thread angles are 0.011° and 0.632°, respectively; and the total average deviation is less than 0.08°. For the screw thread pitch, major diameter, minor diameter, and pitch diameter parameter measurement, the deviation of the measurement results between the proposed method and the universal tool microscope (UTM) method is less than 10 μm. It fully proves the effectiveness and accuracy of the method in this paper and, at the same time, shows that the method has good real-time performance and high application significance, which lays a good foundation for the subsequent online thread measurement.

## 1. Introduction

A sucker rod is an important component of sucker rod pumping equipment, which transmits the power of the pumping unit to the downhole oil pump. The fatigue strength and service life of a sucker rod determine the maximum pumping depth and displacement of the whole set of oil pumping equipment. In the process of oil production, a sucker rod is subjected to asymmetric cyclic loads, and the breakage of the sucker rod will seriously affect the production of crude oil and increase the cost of workover operations and the cost of crude oil. The failure parts of a sucker rod are mainly concentrated in the external thread and the thread relief groove, so the processing quality of the thread directly affects the life of a sucker rod [1,2,3].

**Figure 1:**
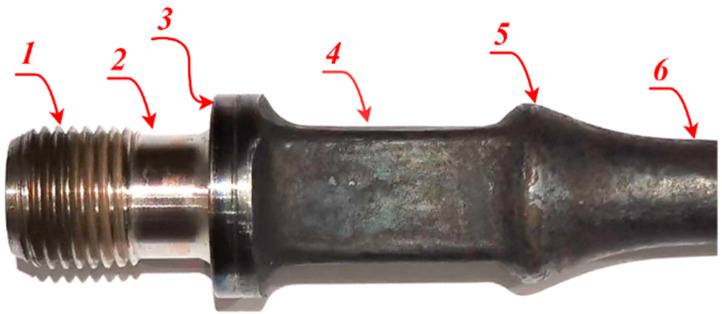
Structure of sucker rod: (1) thread; (2) relief groove; (3) bearing surface; (4) wrench square; (5) transition section; and (6) rod body.

The structure of the end of a sucker rod is shown in Figure 1.

At present, thread parameter measurement usually adopts the method of external-thread go-stop gauge, which is not only time-consuming and labor-intensive, slow in measurement speed, and low in reliability but also prone to problems such as missed inspections and misjudgments. Unqualified sucker rods that are missed or misjudged will cause major production safety accidents and huge economic losses. The development of online rapid precise measurement system has become an urgent problem to be solved, and machine vision technology provides the possibility for the realization of this function.

A variety of approaches have been developed for the precision measurement of external thread. In terms of the measurement method, it is mainly divided into three- and two-dimensional measurements.

Feng et al. proposed the application of corner detection in thread vision measurement [4]. Yang et al. discussed the effect of oil adhesion on the measurement of screw thread with machine vision and put forward the compensation method for the pitch diameter [5]. Lee et al. used machine vision to develop an on-machine thread measurement system for computer numerical control lathe machines and verified the feasibility and accuracy of the on-machine external thread measurement system developed in this study [6]. Man confirmed that the phenomenon of thread profile distortion always exists in measuring screw thread by projection image and proposed the compensation of thread profile distortion in image measuring screw thread [7]. Min designed the measurement method of screw thread geometric error based on machine vision, whose linear precision is less than 10 μm and can be used to detect the comprehensive parameters of screw thread [8]. Senthil et al. proposed the vision measurement of metric screw thread parameters based on orientation invariant feature [9]. Lavrinov et al. presented the laser triangulation 2D scanner signal processing for premium thread pitch measurement [10]. Gadelmawla proposed the computer vision algorithms for measurement and inspection of external screw threads, and the measurement results showed that the maximum difference between the standard and measured values was ±5.4 μm, which shows a good accuracy for measurement [11]. Jing designed a comprehensive measurement system for screw thread parameters based on machine vision, and the feasibility and correctness of this method have been theoretically and practically validated [12]. Wu et al. proposed the thread measurement algorithm research and software development, which does some simple refits to the existing 19JA universal tool-measuring microscope [13]. Shao et al. designed a noncontact thread parameter measuring system based on an array CCD, and the measurement results obtained indicate that this system has advantages such as quick test, high precision, and good repeatability [14]. Hunsicker et al. proposed an automatic vision inspection and measurement system for external screw threads [15].

Antonin proposed a measurement algorithm that adopts the principle of laser triangulation to realize the three-dimensional measurement of the profile parameters of a single cross-section of a screw thread [16]. Farana et al. proposed a method of screw thread measurement using a 3D vision system, which is fast enough to be used on every element in the production line and accurate enough to provide reliable data about the quality of the final product [17]. Sheng et al. presented a new compensation method for measurement of the thread pitch diameter by profile scanning [18]. Tong et al. designed a novel laser-based system for measuring internal thread parameters, and the experimental results show that the measurement accuracy of the thread angle is 2′ and the other parameters are less than 4 μm [19]. Kosarevsky proposed the detection of screw threads in computed tomography 3D density fields, and the described method can be used to automate many operations during screw thread inspection process [20]. Jiang et al. proposed the research on screw thread vision measurement, analyzed and simulated the screw thread’s 3D mathematic model, and, finally, provided a corresponding algorithm to correct the calculation error resulting from the shading effect of backlight projection [21]. Latypov developed an algorithm to detect screw threads in planar point clouds. The described method can be used to automate many operations during screw thread pre-alignment and drastically reduce the operator’s influence on the measurement process resulting in lower measurement times and increased repeatability [22]. Huang et al. developed a laser-based measuring system, which integrated the triangulation laser measuring system and the dual-axis rotary stage for the thread profile of ball screws [23].

Although existing methods and systems can provide high-precision thread detection results, their designs have the following problems in practical application.

(1) At present, the screw thread images in most literature are collected under the ideal conditions of the laboratory, and the structural requirements of the image acquisition system in the complex environment during on-machine measurement are not considered.
(2) Due to the existence of the helical structure in the thread, the position of the thread teeth in the different thread images collected on-machine is uncertain, and the template matching method based on grayscale or shape cannot solve the precise positioning of the thread teeth; therefore, the precise measurement of the thread cannot be realized.
(3) Due to the generation of static electricity, iron filings or dust will adhere to the thread teeth during the threading process. How to design algorithms to effectively remove such noise factors is also the focus of research.

Aiming at the above problems, inspired by the robotic arm in the literature [6], a machine vision system with an adjustable light source device based on a manipulator is designed to solve the first problem above. Based on the characteristics of the thread profile, this paper extended the concept of directional edge detection in the literature [8], and a directional edge detection operator is proposed to solve the problem of precise positioning of the thread teeth. Noise filtering algorithms are developed based on the connected domain to solve the third problem above, and the on-machine high-precision measurement of thread parameters is realized.

## 2. System Setup

### 2.1. Sucker Rod Thread

**Figure 2:**
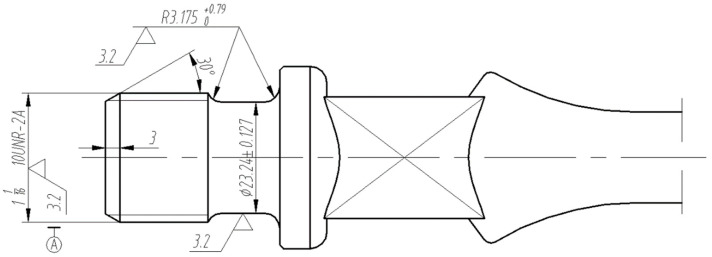
Theoretical geometric dimension requirement of sucker rod.

In practical applications, there are many types of sucker rods. The research object selected in this paper is the model $1\frac{1}{16}$, whose theoretical geometric dimension requirement is shown in Figure 2.

The geometric dimension of the sucker rod thread includes five parameters: the major diameter, minor diameter, effective diameter, pitch, and thread angle. Table 1 lists the measurement standard of the end thread of the sucker rod with model $1\frac{1}{16}$, which is developed by the American Petroleum Institute.

### 2.2. Measurement System

**Figure 3:**
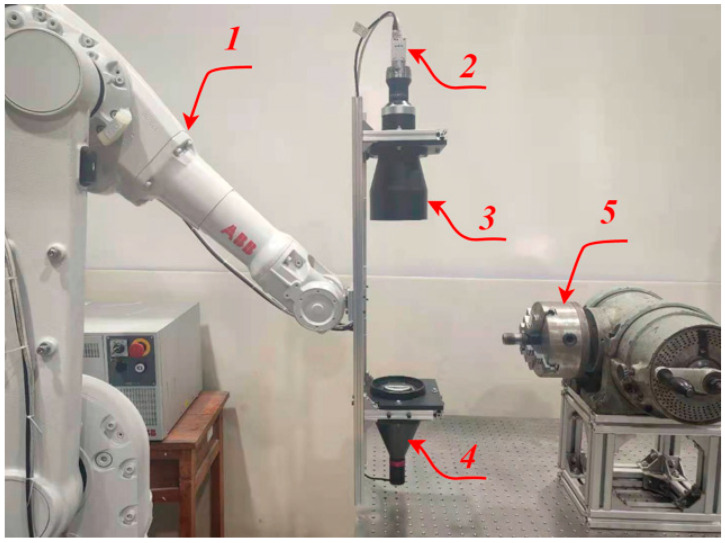
Measurement platform: (1) manipulator; (2) camera; (3) telocentric lens; (4) parallel light; and (5) analog CNC lathe machine tool.

The screw thread measurement system is shown in Figure 3, and it includes a machine vision module, manipulator, and analog CNC lathe machine tool. Among them, the manipulator is a robot produced by ABB, which can realize six degrees of freedom movement and meet the needs of complex working conditions. The machine vision module includes a camera, telocentric lens, and parallel light source.

The measurement based on machine vision mainly includes two parts: the hardware and software parts. The hardware system is responsible for stably and reliably collecting high-quality thread images, involving the selection of light sources, cameras, and lenses, as well as reasonable layout; the software part is responsible for the processing and analysis of thread images, and the accurate calculation of various parameters of the screw thread.

In order to ensure the accuracy of thread dimension measurement, the machine vision module adopts a high-performance industrial camera to capture the thread image at a resolution of 5480 × 3648. Taking full account of the structure and measurement tolerance requirements of the measured screw thread, the parameters of the camera and lens are listed in Table 2.

In order to realize multitype thread measurement, the manipulator can carry the light source device for adaptive light source adjustment. The manipulator motion trajectory planning includes the origin waiting position, internal workpiece position, and motion trajectory. The motion trajectory needs to be taught in advance to avoid collisions between the manipulator and the CNC lathe machine tool.

### 2.3. Principle of Dimension Calculation

Most of the previous literature on thread measurement directly adopts the standard part ratio method [8,11], which is the ideal condition and cannot be applied to the actual production line. In order to improve the robustness of dimensional measurement accuracy, this paper converted pixel coordinates to world coordinates for dimension calculation based on camera calibration parameters.

**Figure 4:**
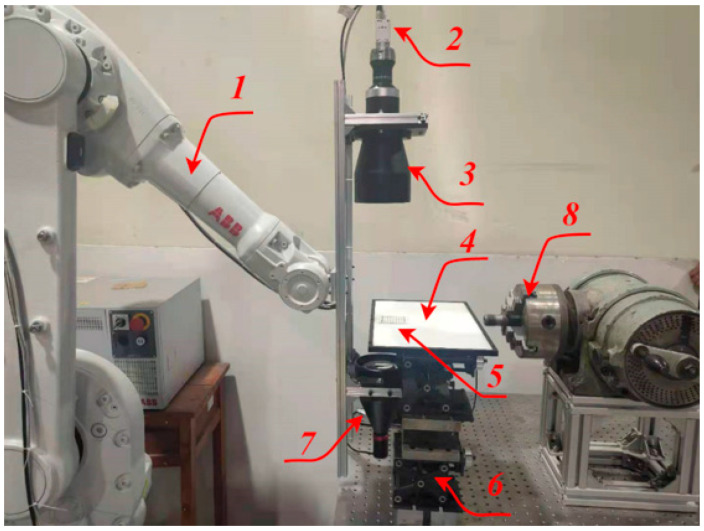
Camera calibration platform: (1) manipulator; (2) camera; (3) telocentric lens; (4) light source plate; (5) calibration plate; (6) height adjustable device; (7) parallel light; and (8) analog CNC lathe machine tool.

The height of the diffuse reflection light source plate 4 is at the same height as the center line of the tested thread by adjusting the height adjustment mechanism 6. On the premise of ensuring that the captured image is clear, 20 pictures of the calibration plate 5 in different positions in space are collected, and the first picture is selected as the main world coordinate system. The camera calibration system is shown in Figure 4.

Based on pinhole projection, assuming the measurement plane in the main world coordinate system is $Z_{W}=0$, the perspective projection formula from the world coordinates to the pixel coordinates [24] is as follows:

(1) $$s\left[\begin{array}{c} u\\ v\\ 1 \end{array}\right]=\left[\begin{array}{ccc} \alpha & \gamma & u_{0}\\ 0 & \beta & v_{0}\\ 0 & 0 & 1 \end{array}\right]\left[\begin{array}{c} R\;\;T \end{array}\right]\left[\begin{array}{c} X_{W}\\ Y_{W}\\ 0\\ 1 \end{array}\right]=\left[\begin{array}{ccc} \alpha & \gamma & u_{0}\\ 0 & \beta & v_{0}\\ 0 & 0 & 1 \end{array}\right]\left[\begin{array}{c} r_{1}\;\;r_{2}\;T \end{array}\right]\left[\begin{array}{c} X_{W}\\ Y_{W}\\ 1 \end{array}\right]=M_{in}M_{out}\left[\begin{array}{c} X_{W}\\ Y_{W}\\ 1 \end{array}\right]=H\left[\begin{array}{c} X_{W}\\ Y_{W}\\ 1 \end{array}\right]$$

In the equation, (u,v) is the coordinate in the pixel coordinate system, and $\left(X_{w},Y_{w},0\right)$ in the main world coordinate system. $M_{in}=\left[\begin{array}{c} \alpha \;\;\;\;\gamma \;\;\;\;u_{0}\\ 0\;\;\;\;\beta \;\;\;\;v_{0}\\ 0\;\;\;\;0\;\;\;\;1 \end{array}\right]$ represents the intrinsic camera parameters, and $\left[\begin{array}{cc} R & T \end{array}\right]$ represents the extrinsic camera parameters. H is the product of the above two, which is a three-order matrix. s is the scale factor, and R and T denote the rotation and translation matrixes, respectively.

The result of the camera calibration distortion parameter is as follows:

(2) $$\left[k_{1},k_{2},p_{1},p_{2},k_{3}\right]=\left[0.00164,-0.0513,0.0117,0.0141,-0.0482\right]$$

The above distortion parameter results prove that the distortion of telecentric imaging is small, which ensures the accuracy of visual dimension measurement.

The result of the internal parameter matrix obtained by the camera calibration is as follows:

(3) $$M_{in}=\left[\begin{array}{ccc} 126105 & 0 & 3486\\ 0 & 126137 & 2167\\ 0 & 0 & 1 \end{array}\right]$$

The result of the external parameter matrix obtained in the main world coordinate system by the camera calibration is as follows:

(4) $$M_{out}=\left[\begin{array}{ccc} 0.056 & -0.998 & -17.407\\ 0.998 & 0.056 & -12.653\\ -0.004 & 0.009 & 1498.156 \end{array}\right]$$

Thus, the 3-order matrix H can be obtained as follows:

(5) $$H=\left[\begin{array}{ccc} 7088.055 & -125869.124 & 3.027e6\\ 125924.583 & 7128.754 & 1.65e6\\ -0.004 & 0.009 & 1498.156 \end{array}\right]$$

Given the coordinate of the pixel point, multiply the inverse matrix of *H* to the left, and then obtain the corresponding world coordinate by de-homogenization.

(6) $$\left[\begin{array}{c} X\\ Y\\ Z \end{array}\right]=\frac{1}{s}\left[\begin{array}{c} X_{W}\\ Y_{W}\\ 1 \end{array}\right]=H^{-1}\left[\begin{array}{c} u\\ v\\ 1 \end{array}\right]$$

(7) $$\left[\begin{array}{c} X_{W}\\ Y_{W} \end{array}\right]=\left[\begin{array}{c} X/Z\\ Y/Z \end{array}\right]$$

After solving formula (7) to obtain $X_{w}\;\mathrm{and}\;Y_{w}$, the real space point $\left(X_{w},Y_{w},0\right)$ in the main world coordinate system is obtained. The screw thread dimension measurement is the distance between these real points.

### 2.4. MPEE and Measurement Uncertainty

**Figure 5:**
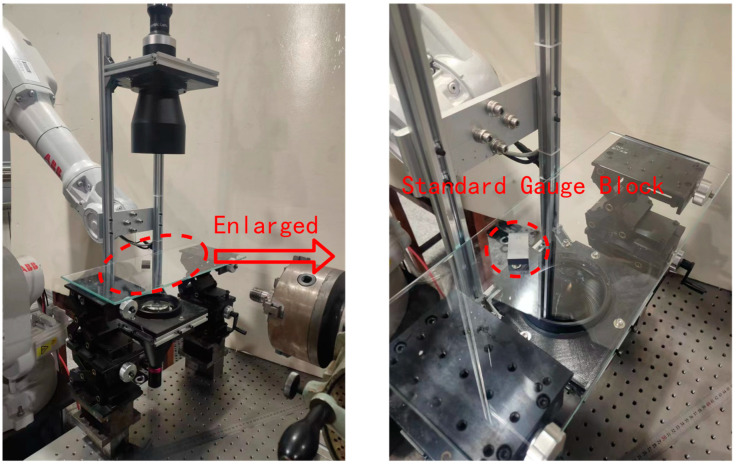
Measurement experiment process.

For a high-precision measurement system, there are many factors that affect the measurement error, which need to be verified by experiments. In order to verify the validity of the above-mentioned visual measurement system, the maximum permissible error for length measurement (MPEE) and the measurement uncertainty of the measurement system are given. In this paper, the following verification scheme was designed with reference to the MPEE calculation process of CMM and the uncertainty of length measurement in the literature [25]. In this paper, three standard length gauge blocks and three angle gauge blocks were selected to realize the calculation of MPEE of this vision system. The three length gauge blocks are all grade I precision gauge blocks; the theoretical values are 5 mm, 8 mm, and 20 mm, respectively, and the accuracy deviations are ±0.2 μm, ±0.2 μm, and ±0.3 μm, respectively. The theoretical values of the three grade I angle gauge blocks are 45°, 60°, and 75°, respectively, and the accuracy deviations are all ±10’’. Because none of the measuring equipment in our laboratory can realize the true value measurement of the above-mentioned length gauge blocks and angle gauge blocks, this paper considered that the theoretical value of each gauge block is its true value. The measurement experiment process is shown in Figure 5.

**Figure 6:**
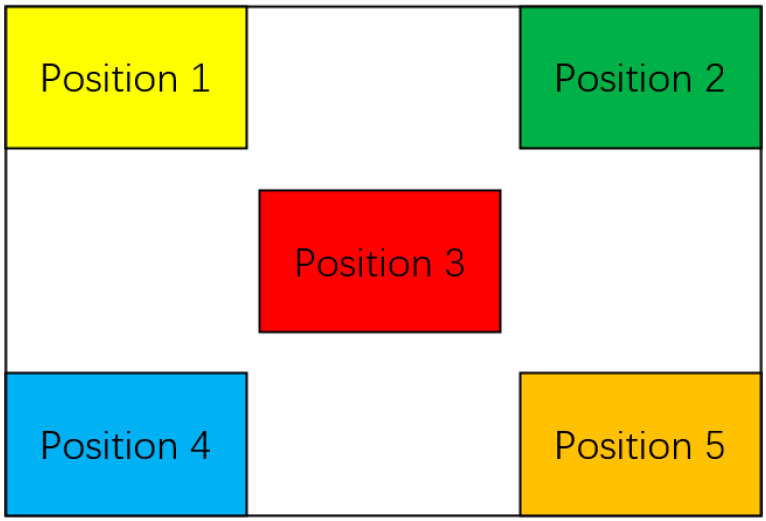
Five positions in the camera’s field of view.

**Figure 7:**
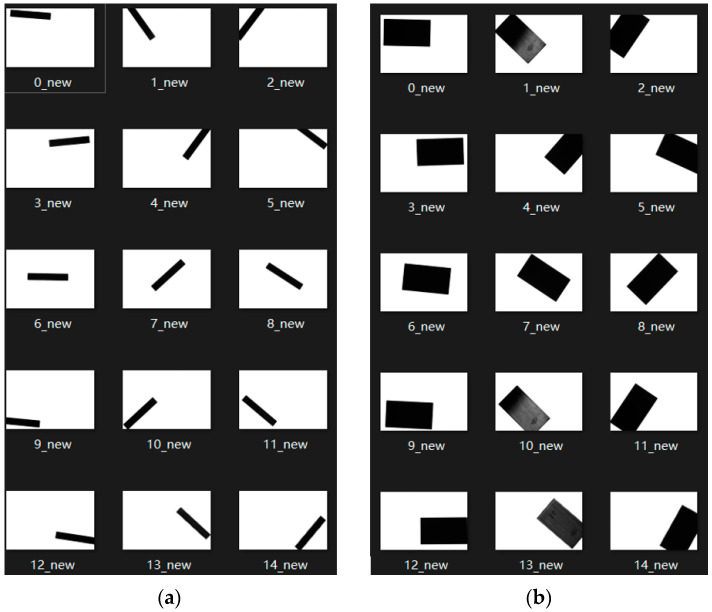
Acquired images of length gauge blocks: (**a**) 5 mm length gauge block; (**b**) 20 mm length gauge block.

**Figure 8:**
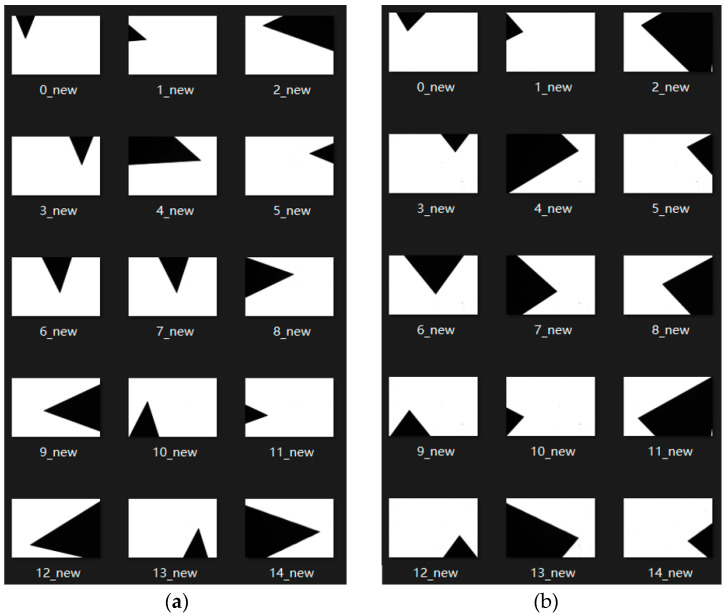
Acquired images of angle gauge blocks: (**a**) 45° angle gauge block; (**b**) 75° angle gauge block.

Each of the above length gauge blocks is placed in five different positions, and three different poses are randomly assigned to each position. For the length measurement experiment, a total of 3 × 5 × 3 = 45 images were collected. The above same process was used in the angle measurement experiment, and 45 angle gauge block images of angle calibration were also obtained. The five positions in the camera’s field of view are shown in Figure 6. The acquired images of the length and angle gauge blocks are shown in Figure 7 and Figure 8, respectively.

After obtaining the collected image of the standard gauge block, based on the camera calibration parameters obtained above, the canny edge detection algorithm is used to realize the visual measurement of the length and angle. The measurement results are shown in Table 3.

As shown in the above table, after measurement and calculation, the MPEE of the visual measurement system in this paper is $30+\frac{L}{10}$ μm, and the accuracy deviation of the angle is 60′′. The measurement results fully verify the validity and accuracy of the measurement system in this paper.

## 3. Algorithm and Principles

**Figure 9:**
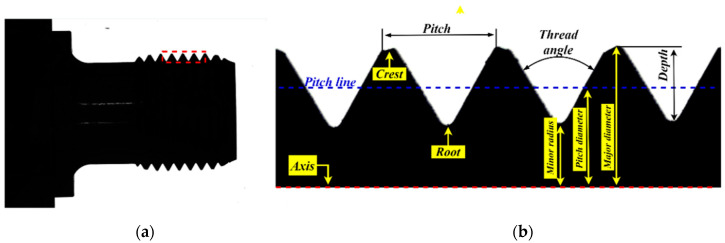
External thread of sucker rod: (**a**) overall image; (**b**) local image of thread.

The actual captured thread image and nomenclatures of thread teeth are shown in Figure 9a,b, respectively. The screw thread dimension measurement in this study measures the thread angle; pitch; and minor, major, and pitch diameters in Figure 9b.

**Figure 10:**
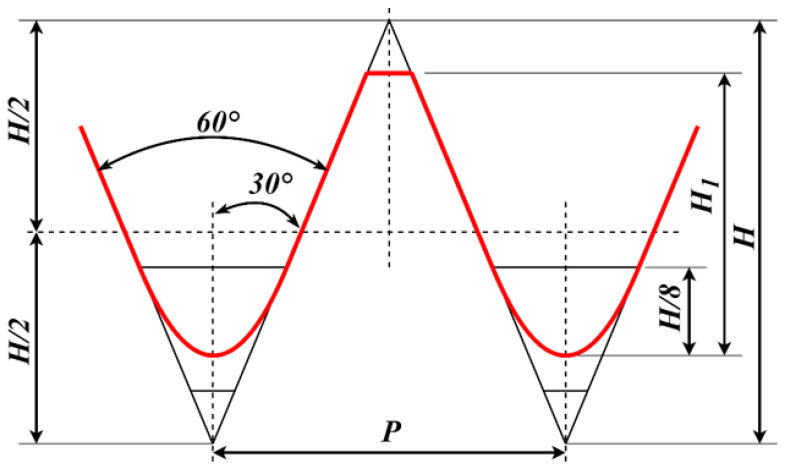
Basic specification of ISO thread [26].

The basic specification of the screw thread is shown in Figure 10.

**Figure 11:**
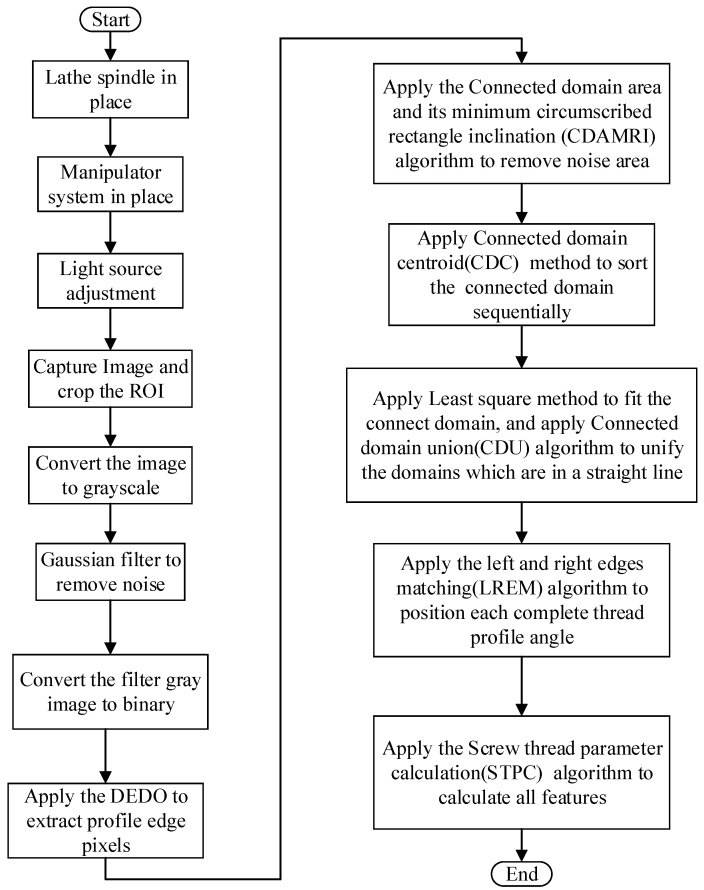
Procedure to measure thread dimensions.

The procedure to measure the thread dimensions is shown in Figure 11.

The procedure of the screw thread dimension measurement includes light source device adjustment, image preprocessing, thread teeth positioning, thread image analysis, and dimension measurement. The screw thread dimension measurement starts from the positioning of the spindle of the CNC lathe to determine the position of the thread workpiece to be measured; and then the manipulator carries the light source device for lighting; after that, screw thread images were captured through the vision module. In order to improve the contrast between the screw thread profile and the background in the image, the method of back-to-parallel light source illumination is adopted, combined with a high-intensity light source, to obtain a clear and sharp thread profile, so as to quickly and easily realize subsequent image processing and analysis.

In order to improve the screw thread dimension measurement speed, the original screw thread image is cropped to obtain the region of interest (ROI) by fixed pixel row and column coordinate position because the position of the threaded workpiece to be tested is almost unchanged. Next, we perform image grayscale, pixel smoothing filtering, and OTSU binarization image preprocessing operations on the ROI area. The obtained binarized image retains the core contour information of the screw thread and then uses the custom directional edge detection operators (DEDOs) to detect the left and right edges of the screw thread. After that, we use the connected domain area and its minimum circumscribed rectangle inclination (CDAMRI) algorithm to filter out the noise area such as dust and oil. Then, in order to facilitate the pairing calculation of the left and right edges of the screw thread, the left and right contour edges of the screw thread are sequentially sorted from top to bottom and left to right based on the connected domain centroid (CDC) method. Actually, on account of the obstruction of dust and oil, the original thread profile edge may be divided into several isolated connected domains after edge detection by DEDO; we design the connected domain union (CDU) algorithm to judge whether the adjacent connected domains are collinear; and then we perform straight-line fitting on each independent unified connected domain and use the left and right edges matching (LREM) algorithm to position each complete thread. Finally, we apply a screw thread parameter calculation (STPC) algorithm to obtain the measured parameter results. The principles of the key algorithms are explained as follows.

### 3.1. Image Processing

After cropping the original image to obtain the ROI, the three-channel original image is grayed by the weighted average method to obtain a single-channel image [27], which improves the subsequent image processing speed. Because the screw thread dimension measurement system is performed on-machine, the actual captured image, which is disturbed by noise, is filtered by a three-order Gaussian convolution kernel [28]. After that, the OTSU [29] with adaptive threshold is used to realize the binarization of the image, and the obtained binarized image data are simple in content and convenient for subsequent image processing and, at the same time, retain the core profile information of the thread to ensure the measurement accuracy.

### 3.2. Directional Edge Detection Operator (DEDO) for Screw Thread Positioning

The premise of obtaining the accurate measurement of screw thread parameters is to realize the accurate positioning of the screw thread. In the actual production process, the position and orientation of the camera in the vision module are fixed, and the spindle of the CNC lathe machine tool rotates with the screw thread to be measured. Due to the characteristics of the helical structure of the screw thread, the positions of the screw thread profile are uncertain when capturing the screw thread image at different times. This means that precise positioning of thread teeth cannot be achieved by template matching. Although there are many papers on the screw thread dimension measurement, very few papers mention the problem of automatic positioning of thread teeth. The corner detection method mentioned in [13] is used to separate the straight-line boundary and bottom arc of the thread teeth. The DP algorithm in [30] is used to approximate the thread profile, but not single thread teeth are positioned in the thread image, which makes automatic measurement of sucker rod end threads impossible. In this paper, according to the prior knowledge of the measured thread profile angle, a directional edge detection operator (DEDO) was specially designed to realize the edge detection of a specific angle in the screw thread image, so as to realize the high-precision positioning of the thread teeth.

#### Principle of DEDO

**Figure 12:**
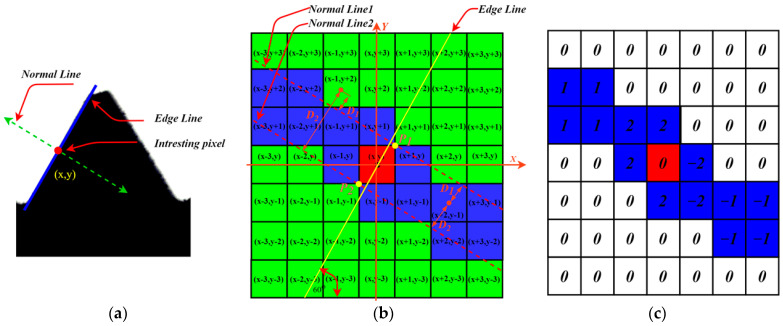
Operator principle: (**a**) edge of thread profile; (**b**) weight calculation principle of kernel; (**c**) directional edge detection kernel.

The calculation principle of the DEDO’s convolution kernel is shown in Figure 12. The standard value of the measured thread angle is 60°. In image edge detection, the normal direction of the edge line is the direction in which the gray value of the pixel changes the fastest. In order to enable the convolution kernel to achieve directional edge detection, it is supposed that the weight of each position in the convolution kernel is related to the distance from the center point and the distance $D_{1},D_{2}$ in Figure 12b.

As shown in Figure 12b, the size of the convolution kernel is 7 × 7, and the center point of the convolution kernel is the origin of the coordinate system. The yellow line is the straight line to be detected at a given angle, and the two red dotted lines are the normal lines of the edge line passing through points P1 and P2. $D_{1}$ and $D_{2}$ are the distances from each position point in the convolution kernel to the two normal lines.

The theoretical functional relationship between weight and distance needs to be further studied and explored in the future. For the convolution kernel of 60° directional edge detection, the weight assignment algorithm is given in this paper as follows:

(8) $$f\left(x,y\right)=\{\begin{array}{cc} 0 & D_{1}+D_{2}\geq Thred1\\ sgn\left(a_{e}x+b_{e}y+c_{e}\right) & \sqrt{x^{2}+y^{2}}\geq Thred2\;and\;D_{1}+D_{2}<Thred1\\ 2*sgn\left(a_{e}x+b_{e}y+c_{e}\right) & \sqrt{x^{2}+y^{2}}<Thred2\;and\;D_{1}+D_{2}<Thred1 \end{array}$$

In the equation, $a_{e},b_{e},c_{e}$ are the coefficients of the edge line equation, and sgn(x) is the signum function.

**Figure 13:**
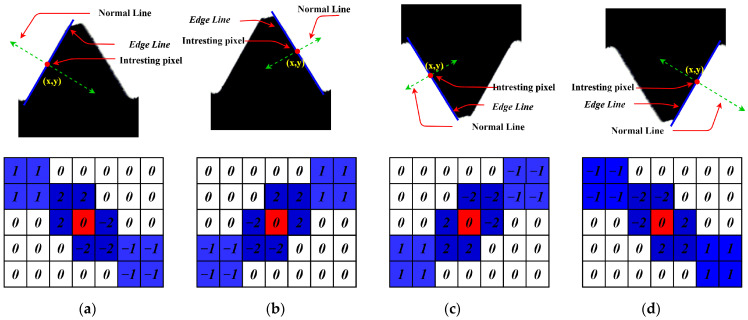
Four DEDOs: (**a**) left edge DEDO of upper thread profile; (**b**) right edge DEDO of upper thread profile; (**c**) left edge DEDO of lower thread profile; (**d**) right edge DEDO of lower thread profile.

Based on the above algorithm principle, the convolution kernel weights are shown in Figure 12c assuming Thred1=2 and $Thred2=\sqrt{2}$. Similarly, all directional edge detection convolution kernels of the thread teeth can be designed, which are shown in Figure 13.

As shown in Figure 13, there is a symmetric relationship between the convolution kernel weights. In fact, (b), (c), and (d) in Figure 13 can be obtained by flipping the left and right or up and down of (a).

**Figure 14:**
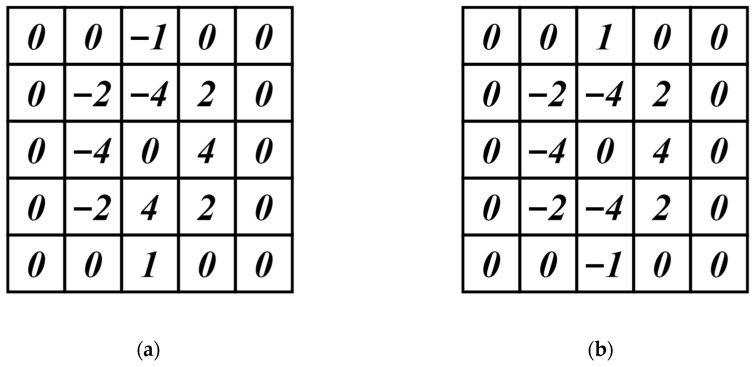
Directional Sobel operator: (**a**) 67.5° direction template; (**b**) 112.5° direction template.

**Figure 15:**
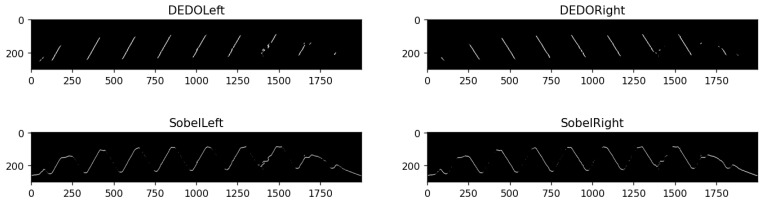
Comparison of edge detection with DEDO and Sobel operator.

The Sobel operator [31] is a widely used edge detection operator in image processing, which has the characteristics of simple principle and fast operation speed. It has been extended to realize edge detection in eight directions. In order to highlight the superiority of the custom DEDOs, we compare the 67.5° and 112.5° convolution kernels in the eight-direction Sobel operator. The 67.5° and 112.5° Sobel convolution kernels are shown in Figure 14, and the results of edge detection with DEDO and the Sobel operator are shown in Figure 15.

As shown in Figure 15, the custom DEDOs increase the weight of the specific directional edge while reducing the weight of the edges in other directions, and the image edge was obviously more concise and precise, which is more favorable for the edge extraction analysis. By comparison, it is found that the 67.5° and 112.5° Sobel operator detects more edge information, which includes the crest and root part of the screw thread, rather than a complete straight line, which interferes with the straight-line fitting of the edge. Thus, the effectiveness and superiority of the proposed DEDO operator are significantly demonstrated.

### 3.3. Noise Area Filtering Based on Connected Domain Area and Its Minimum Circumscribed Rectangle Inclination (CDAMRI)

The edge image detected by DEDOs is affected by dust and oil, and the contour edges of the thread teeth can be segmented into multiple connected domains. At the same time, the edge image may contain small discrete noise domains, which obstruct subsequent image analysis and processing. Firstly, based on the connected domain, the small discrete noise domains whose area is less than the set threshold are deleted. Then, the image is dilated to connect the originally collinear discrete connected domains. Because the result of the image dilation operation is controlled by the structural elements, in order to ensure the accuracy of thread dimension measurement, the structural elements need to expand the edge image as far as possible in the direction of the edge straight line but keep the same as far as possible in the direction of the normal line. These two requirements are exactly the functions of the above DEDOs. Thus, this paper repeatedly used the DEDOs to perform the dilation operation.

**Figure 16:**
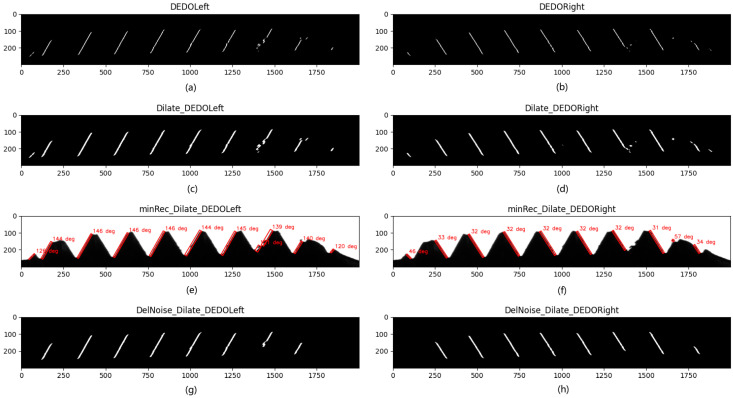
Results of image dilation and filtering out noise: (**a**) the left edge detection with left DEDO; (**b**) the right edge detection with right DEDO; (**c**) the dilation result of the left edge detection; (**d**) the dilation result of the right edge detection; (**e**) the result of the inclination angle of the minimum circumscribed rectangle of each connected domain in the left edge dilation image; (**f**) the result of the inclination angle of the minimum circumscribed rectangle of each connected domain in the right edge dilation image; (**g**) the left edge image after filtering out the noise-connected domains based on the inclination angle; (**h**) the right edge image after filtering out the noise-connected domains based on the inclination angle.

The results of image dilation are shown in Figure 16. Compared with the original edge image, the connectivity of the image is greatly improved. At the same time, it is only limited to the edge straight-line direction, which ensures the accuracy of the straight-line fitting. For other noise-connected domains, after dilation operation, the area and direction are also enhanced to a certain extent. At this time, it can be further filtered out by the area of the connected domain [32] and the angle between the long side of the minimum circumscribed rectangle of the connected domain and the X-axis.

**Figure 17:**
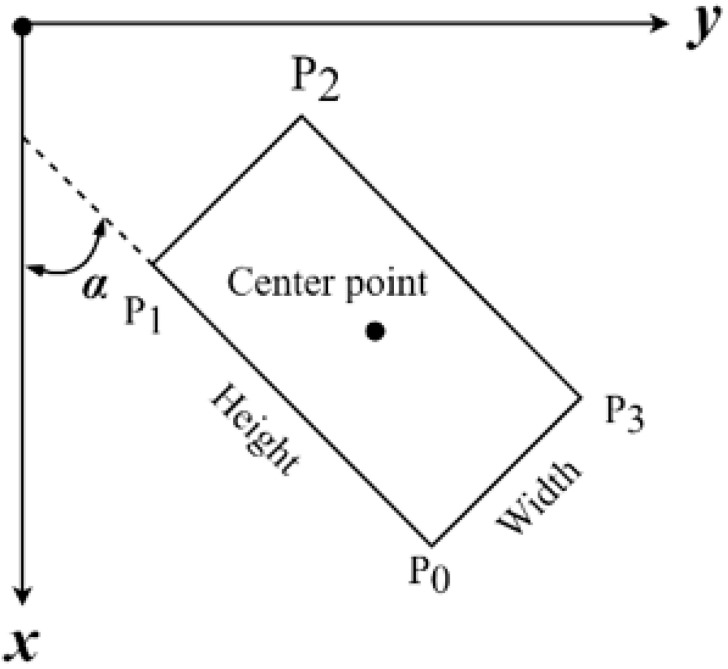
Schematic diagram of minimum circumscribed rectangle inclination angle.

The minimum circumscribed rectangle inclination angle is shown in Figure 17. Let the rectangle P0P1P2P3 be the minimum circumscribed rectangle of a certain connected domain, and define the angle α between the long side P0P1 and the X-axis as the inclination of the rectangle P0P1P2P3. The angle α threshold of the left and right edges is defined as 150° and 30°, respectively. The inclination results of connected domains are shown in Figure 16e,f, and the results after filtering the noise-connected domains are shown in Figure 16g,h.

As shown in Figure 16, based on the CDAMRI algorithm, the noise-connected domains are well filtered out, and only the thread profile edge information we are interested in is retained, which greatly facilitates the subsequent image analysis and processing and improves the dimension measurement accuracy.

### 3.4. Connected Domain Sorting Algorithm Based on Connected Domain Centroid (CDC)

Before realizing the accurate positioning of the thread teeth, it is necessary to sort the connected domains to ensure that the connected domains of the left and right edges of the thread teeth follow the same marking sequence rules. In this paper, the connected domain was marked from top to bottom and from left to right. Considering that the edge lines on the same side of the thread are approximately parallel, the centroids of the connected domains can be used to judge their positional relationship. Common connectivity methods include four-connectivity and eight-connectivity. Based on the eight-connectivity method, this paper used depth-first search (DFS) [33] to locate each connected domain in the image and solve its centroid.

Generally, the centroid calculation formula is as follows:

(9) $$\bar{x}=\frac{\iint_{D}x\rho \left(x,y\right)d\sigma }{\iint_{D}\rho \left(x,y\right)d\sigma },\bar{y}=\frac{\iint_{D}y\rho \left(x,y\right)d\sigma }{\iint_{D}\rho \left(x,y\right)d\sigma }$$

For a two-dimensional image, it is an ideal uniform object, and the pixel coordinate points are discrete. The above formula can be transformed into a formula, that is, the average value of each coordinate point of each pixel point in the connected domain.

(10) $$\bar{x}=\frac{\sum_{1}^{n}x_{i}}{n},\bar{y}=\frac{\sum_{1}^{n}y_{i}}{n}$$

**Figure 18:**
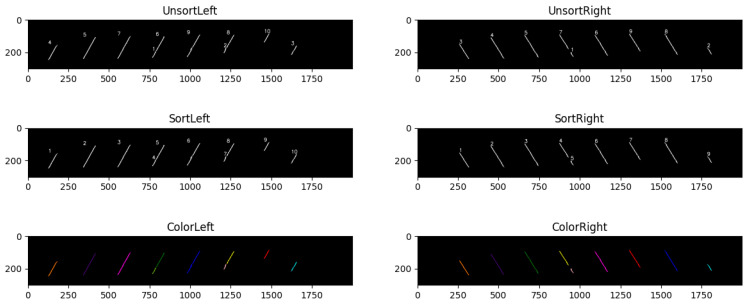
Connected domain sorting results.

Figure 18 shows the results of connected domain sorting. As shown in Figure 18, the sequential sort of the connected domains is well-realized by applying the CDC algorithm, which provides convenience for the subsequent image analysis and dimension measurement.

### 3.5. Connected Domain Union (CDU) Algorithm

The above connected-domain-sorting results show that under the influence of electrostatic dust and oil in the field production environment, even after morphological dilation operations, the screw thread edge may be divided into multiple discrete connected domains. As shown in Figure 12, there are 14 connected domains on the left edge, which actually has only eight complete straight lines. Thus, it is necessary to perform a unified operation on the above sorted connected domains and unify the discrete connected domains that belong to the same thread edge as a new connected domain. Straight-line fitting is performed on each of the above connected domains in order, and the degree of coincidence of the two straight lines before and after can be judged. When the degree of coincidence is greater than the set threshold, it means the two adjacent connected domains before and after belong to the same straight line. Considering that the edge lines on the same side of each thread are approximately parallel, the inner product between the straight-line coefficients cannot be directly used for judgment. On the other hand, the intercept of the straight line and the Y-axis is significantly affected by the size of the image. Even if the angle between the two adjacent straight lines is small, if the image pixels are large, the difference between the intercepts of the two straight lines is large enough, even exceeding the pitch of the screw thread. In this paper, the connected domain centroid data obtained in the CDC algorithm were used to judge whether the two adjacent connected domains belong to the same profile edge of the thread teeth by judging the distance from the connecting centroid to the previous straight line. The pseudocode of the CDU algorithm is shown in Algorithm 1.**Algorithm 1** Connected Domain Union (CDU)**Input:**Contours: Connected domain lists, ret: remove the connected domain whose area is smaller than ret **Output:**lineParas: the fitting line parameters of the unified connected domain, centroids: the centroid of unified connected domain 1: function UnionFitline (Contours, ret) 2: lineParas←[] 3: formerContour←[] 4: centroids←[] 5: formerlinePara←0 6: for contour in Contours**do** 7: linePara← Apply LS algorithm to fit the contour 8: contourcentroid← Calculate the Geometric Moment of the contour to get the centroid 9: distacne← Calculate the distance from contourcentroid to formerlinePara 10: if formerContour is not None and distance<ret then 11:   contour←Unify the formerCoutour and contour 12:   linePara←Apply LS algorithm to fit the contour 13:   contourcentroid← Calculate the Geometric Moment of the contour to get the centroid 14:   lineParas.pop() 15:   centroids.pop() 16: **end if** 17: lineParas.append(linePara) 18: centroids.append(centroid) 19: formerlinePara ←linePara 20: formerContour←contour 21: **end for** 22: return linParas, centroids **end function**


**Figure 19:**
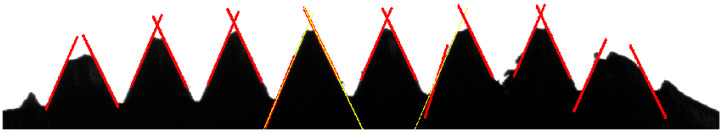
Results of line fitting.

Straight lines of the new connected domains, which are obtained by the CDU algorithm, are fitted with the least squares method [34], and the results are shown in Figure 19.

As shown in Figure 19, the yellow straight line is the fitting result of the new connected domain, and the red line below it is the fitting straight line of a certain connected domain. It can be found that the fitting result of the new connected domain better fits the edge information, which proves the necessity of performing the unified operation of segmented connected domains and ensures the measurement accuracy of thread dimension.

### 3.6. Left and Right Edges Matching (LREM) Algorithm

**Figure 20:**
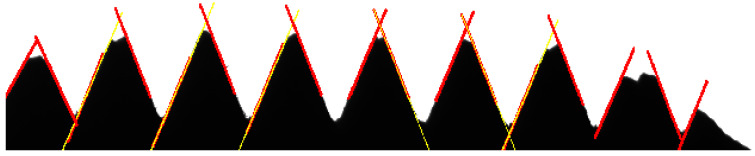
Inconsistent number of left and right edges.

The thread profile edge detection results are significantly affected by on-site dust and oil pollution. If the left and right edges of the same thread teeth are polluted to different degrees, the number of detected left and right edges of the thread will be inconsistent. The inconsistent number of left and right edges is shown in Figure 20. In Figure 20, the number of left edges is one more than that on the right side, so it is necessary to judge whether the left and right edges are matched.

The left and right edges matching (LREM) algorithm can be achieved by calculating the distance of the centroid of the connected domain. The pseudocode is shown in Algorithm 2.**Algorithm 2** Left and Right Edges Matching (LREM)Input: leftCentroids: the centroids of the left profile of thread; rightCentroids: the centroids of the right profile of thread; leftlineParas: the fitting line parameters of the left profile of the thread; rightlineParas: the fitting line parameters of the right profile of thread; ret: the left and right profile are matching when the distance of Y coordinate of left and right centroid is not larger than ret Output: anglelists: the angle lists of left and right profiles of thread; pointCoords: the intersection point lists of the left and right profiles of thread 1: function PixelEdgeMatching(leftCentroids, rightCentroids, leftlineParas,     rightlineParas, ret) 2: anglelists ← [] 3: pointCoords←[] 4:  leftSize←leftCentroids.size 5:  rightSize←rightCentroids.size 6: leftId←0 7: rightId←0 8: while leftId<leftSize and rightId<rightSize**do** 9:  leftY←leftCentroids[leftId].Y 10:  rightY← rightCentroids[rightId].Y 11:  if leftY>right Y**then** 12:   rightId> rightId+1 13:   continue 14:  else if rightY−leftY>ret**then** 15:   leftId←leftId+1 16:   continue 17:  **else** 18:   leftP ←leftlineParas[leftId] 19:   rightP←rightlineparas[rightId] 20:   angle, pCoord ← Calculate the angle-off and intersection point coordinates in use of leftP and rightP 21:   anglelists.append(angle) 22:   pointCoords.append(pCoord) 23:   leftId←lefteId+1 24:   rightId←rightId+1 25:  **end if** 26: **end while** 27: return angle lists, pointCoords 28: **end function**

**Figure 21:**
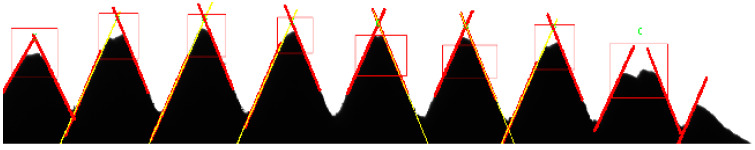
Positioning of thread teeth based on LREM algorithm.

Figure 21 shows the precise positioning of the thread teeth with the LREM algorithm. After that, based on the least squares method, the parametric equation of the fitted straight line can be obtained, and then the thread teeth profile angles, as well as parameters such as the pitch and major diameter, can be obtained.

## 4. Experiments and Discussion

### 4.1. Measurement of Standard Angle Gauge Block

**Figure 22:**
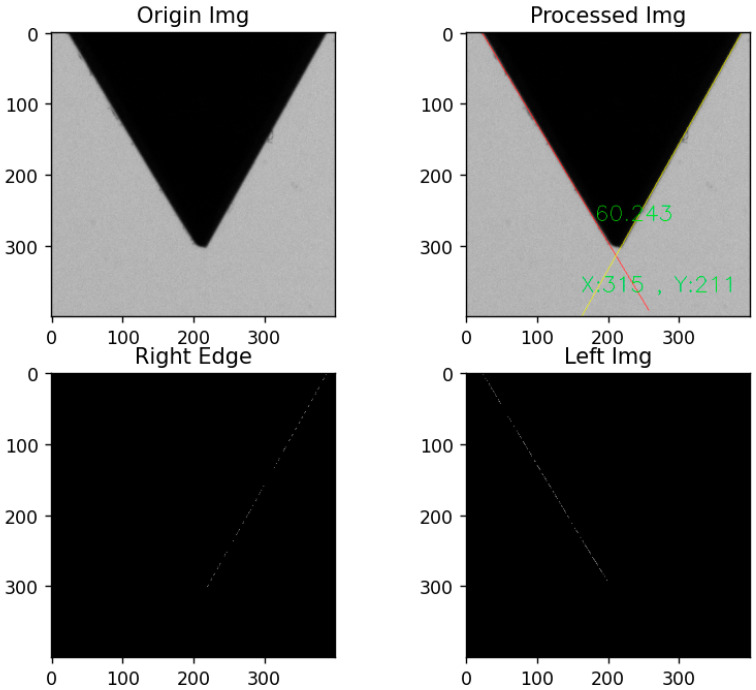
Detection and measurement result of standard gauge block.

To verify the accuracy of the DEDOs, a standard 60° angle gauge block is tested. The captured image is preprocessed according to the above image preprocessing methods in Section 3.1, and then the directional edge detection is performed on the binarized image by applying the DEDOs. The actual gauge block angle is 60°, and the angle between the left and right sides and the horizontal line is approximately 60° and 120°, respectively. The obtained detection effect and actual measurement angle result are shown in Figure 22.

As shown in Figure 22, the DEDOs accurately detect the edge line at the specified angle and calculates the included angle of the gauge block, which deviates from the standard value by less than 0.3° under the condition with noise such as dust, which proves the effectiveness and accuracy of the DEDO.

### 4.2. Screw Thread Parameters Calculation (STPC) Algorithm

**Figure 23:**
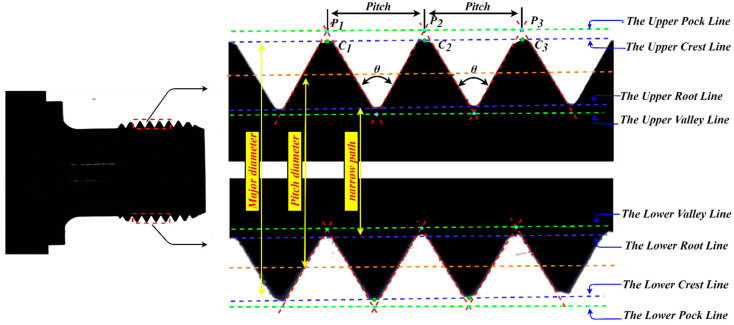
The measurement diagram of the screw thread parameters.

The measurement diagram of the screw thread angle, pitch, major diameter, minor diameter, and pitch diameter parameter measurement is shown in Figure 23.

Due to the generation of static electricity, iron filings or dust will adhere to the thread teeth during the threading process; dimension measurement by the distance between pixel points alone cannot guarantee the accuracy and stability of the measurement. Thus, this paper proposed to use the distance between the fitted straight lines to measure the size so as to ensure the accuracy and robustness of the measurement. As shown in Figure 23, the upper peak point $P_{1},\;P_{2},P_{3}$ is the intersection point of the fitting line between the left and right edges of the upper part of the screw thread, and the upper peak line specifically is the fitting line of the upper peak points $P_{1},\;P_{2},P_{3}$. Similarly, the upper crest line is the fitting line of the upper crest points, which are the intersection points between the thread crest profile and the vertical line through the peak points. Each peak point has its corresponding crest point. In the same way, other fitted straight lines, such as the lower peak and crest line, can be obtained.

#### 4.2.1. Screw Thread Angle

The screw thread angle is the angle between the left and right profile edges of the thread. After the algorithm analysis in the third section, the least squares method can be used to obtain the left and right thread edge fitting straight-line parameters, including the straight-line slope k and the intercept b on the Y-axis. The screw thread angle θ is given by

(11) $$\theta =arctan\left|\frac{k_{2}-k_{1}}{1+k_{1}k_{2}}\right|$$

#### 4.2.2. Pitch

The pitch is the distance between two adjacent thread teeth axes, that is, the distance between two adjacent peak points. The screw threads in the on-machine measurement are subject to many interference factors. In order to ensure the accuracy of the thread pitch measurement, the thread pitches between adjacent peak points are measured, and the average value is taken as the final thread pitch value.

In the image coordinate system, the unit of the calculated pitch is pixels, which needs to be converted to the world coordinate system. Most of the previous literature on thread measurement directly adopts the standard part ratio method, which cannot be applied to the actual production line. Based on the dimension calculation principle in Section 2.3, this paper converted the pixel points to the world coordinate system one by one and then calculated the distance between adjacent points in the world coordinate system to obtain the actual physical value of the thread pitch.

#### 4.2.3. Major Diameter, Minor Diameter, and Effective Diameter

The major diameter of the screw thread is the distance between the crest line in the upper profile of the screw thread and the crest line in the lower profile of the screw thread. In the same way, the minor diameter is the distance between the upper and lower valley lines, and the effective diameter can be calculated by taking the average of the major and minor diameters. In actual measurement, the upper and lower crest lines cannot be guaranteed to be completely parallel, and the major diameter cannot be directly calculated from the slope and the difference of intercept. Taking the measurement of the major diameter of the thread as an example, in order to ensure the accuracy of the measurement, this paper selected multiple crest points in the upper crest line and calculated the distance of these crest points to the lower crest line. Finally, we take the average value as the major diameter of the thread. The measurement of the minor diameter is the same. It should be noted that, similar with the above pitch measurement method, the pixel coordinates are converted to the world coordinate system to obtain the actual physical size.

### 4.3. Discussion

**Figure 24:**
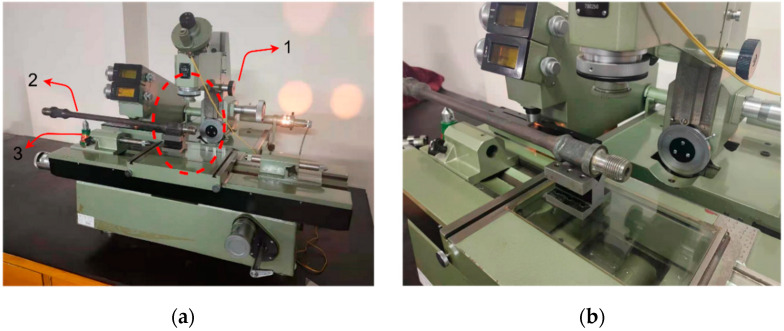
UTM platform: (1) UTM; (2) sucker rod thread; (3) adjustable device. (**a**) Overall display of UTM. (**b**) Partial display of UTM.

In order to verify the validity of the measurement results of the machine vision algorithm proposed in this paper, a 19JA-780/250 universal tool microscope (UTM) shown in Figure 24 was used to measure the screw thread angle, major diameter, minor diameter, and pitch of the end of a sucker rod, and these measurement results are the standard reference values. The indoor temperature is 20 °C during the measurement, the graduation value for the length measurement of this device is 1 μm, and the division value of the 360° angle measurement range is 1’. MPEE: (2.25 + L/40) um; MPEP: 1.5 um. The screw thread measurement platform by applying the UTM is shown in Figure 24.

**Figure 25:**
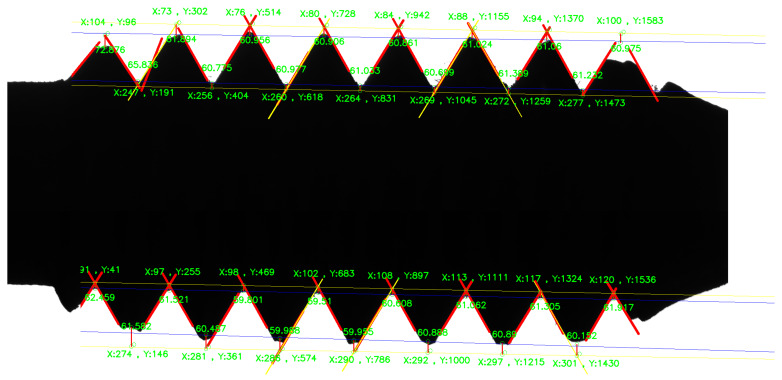
Measurement results of screw thread dimension.

The results of the screw thread parameters measured by machine vision and the UTM are shown in Table 4. The display of measurement results in the screw thread is shown in Figure 25.

It can be shown from Table 3 that

(1) The actual measured value of the tooth profile angle is greater than the theoretical value. After analysis, it is the reason for the real processing and production. As shown in Table 1, although the theoretical standard value of the thread angle is 60°, it has a large qualified interval, from 59.5 to 61.5, and other size parameters also have corresponding size ranges.
(2) The minimum and maximum deviations of the thread angle measured by the proposed method and the UTM method are 0.011° and 0.63°, respectively, and the total average deviation is less than 0.08°, which proves the effectiveness and accuracy of the method in this paper for the thread angle. Moreover, the robustness of the visual measurement method is better than that of the UTM method.
(3) For the screw thread pitch, major diameter, and minor diameter parameter measurement, the deviation of the measurement results of the proposed method and the UTM method is less than 10 μm. At the same time, the stability of the proposed method is better than that of the UTM method, and the effectiveness and accuracy of the visual method proposed in this paper for on-machine thread dimension measurement are significantly demonstrated. On the other hand, the completion time of the thread parameter measurement method proposed in this paper is about 0.13 s, which fully meets the real-time requirements of online thread measurement.

The positioning of each thread teeth and the measurement of each of the thread parameters are shown in Figure 25. In Figure 25, (X,Y) is the coordinate of the intersection point in the pixel coordinate system.

## 5. Conclusions

Most previous studies mainly focused on the off-machine thread dimension measurement and rarely discussed on-machine detection and measurement, which is still not up to the requirements of practical production applications. Thus, this paper studied the on-machine measurement method of sucker rod end screw threads based on machine vision. The procedure of the screw thread dimension measurement includes light source device adjustment, image preprocessing, thread positioning, thread image analysis, and dimension measurement.

(1) CNC machine tools and a manipulator are combined to achieve adjustable light source illumination for high-quality image acquisition of screw threads.
(2) The custom DEDOs based on the prior knowledge of the thread profile edge angle is developed to accurately and reliably detect the left and right edges of the thread teeth for the positioning of the thread teeth.
(3) CDAMR, CS, CD, and LREM algorithms are developed to eliminate the influence and interference of electrostatic dust and oil pollution in on-site measurement so as to achieve high-precision measurement of the thread profile angle, pitch, major diameter, minor diameter, and pitch diameter.
(4) The experimental results show that the maximum and minimum average errors of the thread angles are 0.011° and 0.63°, respectively, and the total average deviation is less than 0.08°. For the screw thread pitch, major diameter, minor diameter, and pitch diameter parameter measurement, the deviation of the measurement results of the proposed method and the UTM method is less than 10 μm. At the same time, the completion time of the thread parameter measurement method is about 0.13 s, which fully meets the real-time requirements of thread online measurement.

In general, the method proposed in this paper achieves high-precision thread parameter dimension measurement under the complex background containing dust and oil pollution, which proves the effectiveness and accuracy of the method proposed. In the future, the measurement accuracy can be further improved by optimizing the camera calibration method and using subpixels. This system has laid a good foundation for the thread on-line measurement of subsequent production lines and has great practical application significance.

## Figures and Tables

**Table 1:** Measurement tolerance of thread with model $1\frac{1}{16}$ (10UNR-2A).

Pitch: 2.54 mm; 10 teeth/inch; Thread Angle: 60			
Major Diameter	Minor Diameter	Pitch Diameter	Thread Angle
∅26.624–∅26.952	∅25.146–∅25.303	≤∅23.835	59.5–61.5

**Table 2:** Inherent parameters of camera and lens.

Index	Parameter Names	Parameters Values
1	Pixels of camera	5480 × 3648
2	Physical size of the camera chip	2.4 μm × 2.4 μm
3	Area of the camera chip	5480 × 2.4 = 13.152 mm 3648 × 2.4 = 8.7552 mm
4	Lens ratio	0.202
5	Visual field	13.152/0.202 = 65.11 mm 8.7552/0.202 = 43.34 mm

**Table 3:** Measurement results of length gauge blocks and angle gauge blocks.

Positions	Length Gauge Blocks			Angle Gauge Blocks		
	5 mm	8 mm	20 mm	45°	60°	75°
Position1	5.016	8.085	20.016	44.985	60.003	75.018
	5.024	8.043	20.013	44.995	60.003	74.904
	5.019	8.016	20.012	45.002	60.004	75.006
Position2	5.017	8.009	20.076	44.988	60.054	75.001
	4.994	8.037	20.026	44.983	60.018	74.998
	5.025	8.016	20.051	44.996	59.995	75.004
Position3	4.982	8.014	20.028	45.012	59.98	74.992
	5.235	8.016	20.066	45.008	60.02	75.015
	5.019	8.089	20.072	45.007	59.998	75.011
Position4	5.022	8.014	20.011	44.994	60.028	75.049
	5.001	8.005	19.981	44.995	59.992	75.006
	5.015	8.067	20.048	45.034	59.995	75
Position5	5.022	8.017	20.017	45.066	60.006	75.001
	5.02	8.013	20.021	45.004	59.995	75.009
	5.017	8.011	20.031	45.045	59.992	75.011
$\bar{x}$	5.029	8.030	20.031	45.008	60.006	75.002
σ	0.058	0.028	0.026	0.023	0.018	0.030
$\mu_{A}$	0.018	0.008	0.008	0.007	0.005	0.009
**MPEE**	(30+L/10) μm L is the measured length, in mm			60′′ $0^{0}–360^{0}$ angle measurement		

**Table 4:** Measurement results of STPC and UTM.

Index	Method	Thread Angle			Pitch1		Maj D	Min D	D
		1	2	3	1	2	---------	---------	---------
1	DEDO	**60.991**	**61.485**	**60.143**	**2.543**	**2.543**	**26.519**	**23.425**	**24.972**
	UTM	61.254	61.282	60.775	2.539	2.538	27.761	23.447	25.104
2	DEDO	**60.711**	**61.425**	**60.578**	**2.531**	**2.531**	**26.625**	**23.449**	**25.037**
	UTM	60.454	61.903	60.885	2.542	2.576	26.811	23.443	25.127
3	DEDO	**60.465**	**61.431**	**60.533**	**2.555**	**2.531**	**26.673**	**23.437**	**25.055**
	UTM	60.627	61.026	61.334	2.541	2.546	26.859	23.466	25.163
4	DEDO	**60.679**	**60.912**	**60.682**	**2.543**	**2.543**	**26.708**	**23.465**	**25.087**
	UTM	60.386	61.676	60.353	2.541	2.538	26.901	23.475	25.189
5	DEDO	**60.694**	**61.326**	**60.199**	**2.542**	**2.533**	**26.733**	**23.441**	**25.087**
	UTM	61.325	61.025	60.253	2.503	2.594	26.845	23.479	25.162
6	DEDO	**60.685**	**60.791**	**60.395**	**2.531**	**2.542**	**26.757**	**23.466**	**25.112**
	UTM	60.584	61.528	60.706	2.537	2.534	26.859	23.502	25.179
7	DEDO	**60.805**	**61.451**	**60.946**	**2.532**	**2.531**	**26.534**	**23.467**	**25.001**
	UTM	60.845	61.335	60.885	2.544	2.539	26.913	23.507	25.211
8	DEDO	**60.395**	**61.478**	**60.733**	**2.543**	**2.542**	**26.632**	**23.468**	**25.05**
	UTM	60.528	61.523	60.854	2.551	2.538	26.821	23.507	25.164
9	DEDO	**60.711**	**61.322**	**60.699**	**2.542**	**2.535**	**26.672**	**23.482**	**25.077**
	UTM	61.213	61.547	60.668	2.537	2.547	26.821	23.464	25.143
10	DEDO	**60.946**	**61.531**	**60.607**	**2.542**	**2.543**	**26.722**	**23.446**	**25.084**
	UTM	60.497	61.548	60.582	2.538	2.543	26.929	23.417	25.173
$\bar{x}$	DEDO	60.708	61.315	60.552	2.540	2.537	26.658	23.455	25.056
	UTM	**60.771**	**61.439**	**60.730**	**2.537**	**2.549**	**26.952**	**23.471**	**25.162**
σ	DEDO	**0.184**	**0.255**	**0.247**	**0.007**	**0.006**	**0.081**	**0.018**	**0.041**
	UTM	**0.362**	**0.276**	**0.303**	**0.012**	**0.019**	**0.287**	**0.029**	**0.031**
$u_{95}$	DEDO	**0.132**	**0.182**	**0.177**	**0.005**	**0.004**	**0.058**	**0.013**	**0.029**
	UTM	**0.259**	**0.197**	**0.217**	**0.009**	**0.014**	**0.205**	**0.021**	**0.022**
